# Climate-Driven Perturbations in Influenza Subtype Positivity Before and After the COVID-19 Pandemic: Observational Study

**DOI:** 10.2196/91124

**Published:** 2026-11-11

**Authors:** Jiajia Lin, Congzhu Li, Lirong Zhang, Jiaxiong Zheng, Yixiao Niu, Di Lin, Yan Che, Zicheng Cao

**Affiliations:** 1 State Key Laboratory of Public Big Data, Guizhou University, Guiyang, People's Republic of China; 2 Engineering Research Center for Big Data Application in Private Health Medicine of Fujian Universities, Putian University, Putian, People's Republic of China; 3 Department of Gynecologic Oncology, Cancer Hospital of Shantou University Medical College, Shantou, People's Republic of China; 4 Shantou University Medical College, 22 Xinling Road, Shantou, Guangdong, 515041, People's Republic of China, 86 15989246139

**Keywords:** seasonal influenza, climatic factors, COVID-19 pandemic, interpretable machine learning, epidemiology

## Abstract

**Background:**

The COVID-19 pandemic significantly altered global influenza transmission patterns. Whether the associations among meteorological conditions, visibility, and influenza subtype (A/H1N1, A/H3N2, and B/Victoria) positivity changed after the pandemic remains unclear. We compared these associations before and after the COVID-19 pandemic in northern China.

**Objective:**

This study aimed to characterize changes in the associations of meteorological variables and visibility with influenza subtype positivity before and after the COVID-19 pandemic and to identify subtype-specific nonlinear patterns and thresholds.

**Methods:**

Using 13 years of influenza surveillance and environmental data, we applied Extreme Gradient Boosting enhanced with Shapley additive explanations (SHAP) to characterize the relative importance and direction of associations between environmental variables and influenza subtype positivity. Generalized additive models (GAMs) were subsequently used to characterize nonlinear association patterns and identify thresholds at which the direction of model-estimated associations changed.

**Results:**

Absolute humidity (AH) remained the most influential environmental factor for A/H3N2 and A/H1N1, accounting for 32.2% and 38.5% of relative feature importance, respectively. Visibility increased in importance after the pandemic, rising from being the fourth to the second most influential environmental indicator. GAM analysis identified thresholds corresponding to points where the direction of the associations changed. For A/H3N2, AH below 4 g/m^3^ was associated with positive SHAP values, whereas higher AH was predominantly associated with negative SHAP values. For A/H1N1, the direction of association changed at an AH threshold of 4.5 g/m^3^ (95% CI 4.0-4.8) in the postpandemic period. Additionally, relative humidity (RH) demonstrated a complex nonlinear association with A/H3N2 positivity, with transition points at 45.1% (95% CI 43.5%-46.1%) and 69.7% (95% CI 69.2%-70.2%) in the prepandemic period and at 48.7% (95% CI 46.7%-50.4%) and 70.6% (95% CI 66.3%-72.6%) in the postpandemic period. RH values within these ranges were associated with negative SHAP values, whereas values outside these ranges were associated with positive SHAP values. In contrast, visibility showed opposing association patterns for the A/H3N2 and B/Victoria subtypes after the pandemic. Visibility below 17.1 km (95% CI 16.3-17.4) was associated with negative SHAP values for A/H3N2, whereas visibility below 18.4 km (95% CI 18.0-18.9) was associated with positive SHAP values for B/Victoria.

**Conclusions:**

AH remained a key environmental determinant of influenza A subtype positivity, while visibility became more important after the pandemic and showed opposing association patterns for A/H3N2 and B/Victoria. The identified nonlinear thresholds highlight subtype-specific environmental associations and may inform influenza surveillance and risk assessment under changing environmental conditions.

## Introduction

Seasonal influenza remains a significant global health concern, causing substantial morbidity and mortality worldwide. The World Health Organization estimates that annual influenza epidemics result in approximately 3 to 5 million cases of severe illness and 290,000 to 650,000 respiratory deaths worldwide [1]. The activity patterns of influenza are influenced by a complex interplay of virological, ecological, and meteorological factors [2,3]. Among these, environmental elements such as temperature, humidity, and air quality play critical roles in modulating viral transmission and survival [4,5]. Traditionally, seasonal influenza has exhibited predictable patterns, with transmission peaks during colder months in temperate regions [6]. However, the COVID-19 pandemic dramatically altered these established patterns of seasonal influenza infection worldwide [7,8]. The implementation of public health and social measures (PHSMs) to combat COVID-19, such as mask wearing, social distancing, and reduced international travel, led to unprecedented changes in influenza transmission dynamics [9,10]. This global disruption presents a unique opportunity to reassess the relationship between environmental factors and influenza transmission in the context of altered human behavior and ecological interactions.

In northern China, the epidemiology of seasonal influenza is characterized by distinct patterns that have undergone significant changes due to the COVID-19 pandemic. Prior to the pandemic, multiple influenza subtypes, including A/H1N1, A/H3N2, and B/Victoria, typically cocirculated during the regular influenza seasons [11,12]. This multisubtype cocirculation pattern was a hallmark of influenza activity in the region. However, with the onset of the COVID-19 pandemic, northern China experienced a dramatic shift in influenza dynamics. Influenza positivity rates plummeted to near-zero levels, ushering in an unprecedented “influenza-free period” that persisted for nearly a year [10,13]. This stark reduction in influenza activity coincided with the implementation of stringent PHSMs to control COVID-19 transmission. As China gradually relaxed its pandemic control measures, a remarkable resurgence of influenza activity was observed. Postpandemic influenza seasons have been characterized by large-scale outbreaks with positivity rates surpassing prepandemic levels [14]. Notably, these postpandemic seasons have predominantly featured single-subtype dominance, a significant departure from the prepandemic multisubtype cocirculation pattern [15]. This shift in influenza epidemiology underscores the need for a deeper understanding of how climatic factors dynamically influence influenza transmission over time, particularly in the context of these altered viral circulation patterns. The region’s distinct climate and population density, coupled with these dramatic changes in influenza activity, make northern China an ideal setting for studying the intricate and evolving relationships between meteorological conditions and influenza transmission [16].

This study aimed to address critical gaps in our understanding of how the COVID-19 pandemic has reshaped the relationship between environmental factors and influenza transmission in northern China. Leveraging 13 years of multisubtype influenza surveillance data and high-resolution meteorological records, we used an interpretable machine learning approach to elucidate these complex interactions. Specifically, we sought to (1) quantify the changing impact of key meteorological variables on the transmission of influenza subtypes A/H1N1, A/H3N2, and B/Victoria before and after the COVID-19 pandemic; (2) identify critical environmental thresholds that signify transitions in influenza risk for each subtype; and (3) assess how the relative importance of different environmental factors in influenza transmission has shifted in the postpandemic era, with a particular focus on the temporal dynamics of these influences.

By addressing these objectives, our study aimed to provide a nuanced understanding of the evolving dynamics between environmental conditions and influenza in the wake of a major global health crisis. The insights gained from this research have significant implications for public health planning and disease prevention strategies. Understanding the shifting relationships between environmental factors and influenza transmission can inform more targeted and effective interventions, potentially reducing the burden of seasonal influenza in northern China and beyond. Moreover, this study contributes to the broader scientific discourse on how large-scale disruptions to human behavior and environmental conditions can reshape infectious disease epidemiology, offering valuable lessons for managing future pandemics and adapting to ongoing global climate changes.

## Methods

### Study Design and Setting

This observational study used longitudinal time series data on weekly influenza subtype positivity and environmental conditions in northern China from 2011 to 2024. All available weekly observations within the defined study period were included in the analysis. The study period was split into a prepandemic period (January 1, 2011-December 25, 2019) and a postpandemic period (November 2, 2022-April 30, 2024) [17,18]. The interval from December 26, 2019, to November 1, 2022, was excluded due to the dynamic zero–COVID-19 policy implemented by the Chinese government during the COVID-19 pandemic. This period saw widespread disruptions in influenza transmission as PHSMs significantly altered the usual seasonal influenza patterns. Analyses were conducted at the regional level, with weekly influenza subtype positivity rates and corresponding environmental measures representing northern China as a whole.

### Data Source and Collection

Weekly influenza surveillance data were extracted from the Chinese National Influenza Center and included the number of laboratory-confirmed cases for 3 distinct influenza subtypes: A/H1N1, A/H3N2, and B/Victoria. B/Yamagata was excluded because no cases of B/Yamagata were reported by the Chinese National Influenza Center in the 2021-2023 cycle [19]. Additionally, the number of samples tested each week was collected. To estimate the weekly positive rate, we calculated the ratio of laboratory-confirmed cases for each subtype to the number of test samples [20,21]. Meanwhile, the influenza season was defined as the period from week 40 of one year to week 20 of the following year, consistent with standard epidemiological practices for seasonal influenza surveillance [19].

Meteorological data were sourced from the Visual Crossing data network [22] and spanned the period from January 1, 2011, to April 30, 2024. Data included variables such as cloud cover (percentage), apparent temperature (degrees Celsius), relative humidity (RH; percentage), sea level pressure (SLP; millibars), solar energy (megajoules per squared meter), solar radiation (watts per squared meter), air temperature (degrees Celsius), UV index, wind direction (degrees), and wind speed (kilometers per hour). Visibility (kilometers) was also included as an environmental indicator reflecting atmospheric conditions. Absolute humidity (AH; grams per cubic meter), defined as the weight of water vapor per unit volume of air, was calculated from air temperature and RH based on known formulas [23]. The diurnal temperature range (DTR; degrees Celsius) was computed as the difference between daily maximum and minimum temperatures. Meteorological data for all northern Chinese cities were aggregated into weekly averages to match the temporal resolution of influenza surveillance data reported by the Chinese National Influenza Center. This generated a unified regional time series that reflected the mean environmental conditions across northern China.

To prepare the influenza and environmental data for analysis, time series preprocessing involved checking for missing values, outlier detection and removal, and normalization to ensure data quality and consistency. Before model construction, Spearman correlation analysis was performed to examine associations between environmental variables and positivity rates of A/H1N1, A/H3N2, and B/Victoria. The correlation coefficients were visualized using heat maps, facilitating the identification of potential relationships between environmental conditions and influenza activity.

### Machine Learning Modeling and Feature Interpretation

We used a machine learning approach with Extreme Gradient Boosting (XGBoost) to model the relationship between environmental variables and the positivity rates of the influenza subtypes. Model performance was assessed using metrics including the coefficient of determination (*R*^2^), root mean squared error, mean squared error, and mean absolute error, with the results provided in Table S1 in Multimedia Appendix 1 [24].

To interpret the model, we used Shapley additive explanations (SHAP), a unified framework that quantifies the contribution of each environmental variable to model predictions [25]. SHAP values for each predictor were calculated, indicating whether each feature made a positive or negative contribution to the model prediction. This allows for a granular, observation-level interpretation of the influence of environmental factors. The SHAP values were derived using the following linear equation:


$$f(x)=\varphi_{0}+\sum_{i=1}^{M}\varphi_{i}$$

In this equation, *f*(*x*) represents the prediction for a given input *x*, φ_0_ is the model’s average prediction, and φ*_i_* represents the contribution of feature *i* to the difference between the actual prediction and the baseline. The total prediction is thus broken down into the sum of the baseline and the contributions from all individual features φ*_i_*, where *M* is the total number of features. SHAP values (φ*_i_*) can either be positive or negative, reflecting how much each feature increases or decreases the predicted outcome. These results were visualized through SHAP summary plots and further stratified by pre- and postpandemic periods to highlight differences in climatic influences.

In addition, generalized additive models (GAMs) were fitted separately for each environmental variable and its corresponding SHAP values to characterize nonlinear patterns in the variable-specific contribution to the XGBoost model output. Model fitting was conducted using the *mgcv* package, which automatically selected the optimal smoothing parameters through generalized cross-validation or restricted maximum likelihood. Nonlinear associations between environmental variables and their SHAP values were described using GAMs. Threshold points were defined as the points at which the fitted GAM curves crossed zero SHAP values, indicating a change in the direction of the model-estimated association between environmental variables and influenza positivity rates. These points were identified using root-finding algorithms. Bootstrap resampling (1000 iterations) was performed to estimate the uncertainty of threshold locations, and the corresponding 95% CIs were calculated.

### Statistical Analysis

All statistical analyses were performed using R (version 4.3.3; R Foundation for Statistical Computing) and Python (version 3.9.0; Python Software Foundation). Continuous variables were summarized as means and IQRs, and differences between 2 groups were evaluated using either the 2-tailed *t* test or Mann-Whitney *U* test as appropriate. The significance level was set at *P*<.05.

### Ethical Considerations

This study used anonymized, aggregated influenza surveillance data provided by the Chinese National Influenza Center. The dataset contained no personally identifiable information and was analyzed in aggregate only. As this study involved secondary analysis of deidentified surveillance data and did not involve direct contact with human participants, ethical review and informed consent were waived because the study involved only secondary analysis of anonymized surveillance data without access to identifiable personal information, in accordance with applicable institutional and national regulations.

All data were deidentified before analysis, and no information that could identify individual participants was available to the investigators. The study findings are reported only at the aggregate level to ensure the protection of participant privacy and confidentiality.

No participant compensation was applicable because no participants were directly recruited for or involved in this study.

## Results

### Overview of Influenza Activity and Meteorological Conditions

During the pre–COVID-19 pandemic period (2011-2019), influenza A subtypes (H3N2 and H1N1) exhibited distinct seasonal patterns with pronounced activity spikes from December to April annually, often cocirculating in most seasons (eg, the winter seasons of 2013, 2017, and 2019). A/H3N2 maintained minor activity even off-season, while A/H1N1 and B/Victoria were primarily confined to the traditional influenza season. B/Victoria showed lower overall positivity rates and less pronounced annual peaks. At the onset of the COVID-19 pandemic (week 10, 2020, to week 1, 2022), influenza activity decreased substantially across all subtypes, with A/H3N2 and A/H1N1 rates dropping to near zero (Figures 1A and 1B). Notably, B/Victoria exhibited unexpected activity during this period, peaking at 32.19% positivity in 2022 (Figure 1C). After the COVID-19 pandemic, particularly after transitioning to routine pandemic management, influenza seasons were characterized by single-subtype predominance with higher intensity than prepandemic levels. From 2022 to 2024, A/H3N2 and A/H1N1 activity rebounded sharply, with postpandemic peaks reaching 33.06% and 32.68%, respectively, significantly higher than pandemic levels (*P*<.001 and *P*=.002, respectively; Figures 1D and 1E). A/H3N2 surpassed prepandemic levels (mean positive rate: 6.30%, SD 9.00% vs 3.54%, SD 5.77%; *P*=.009), while A/H1N1 returned to comparable prepandemic rates. B/Victoria showed significant differences between prepandemic and postpandemic periods (mean positive rate: 1.01%, SD 3.83% vs 3.33%, SD 6.57%; *P*=.003), although pandemic and postpandemic levels did not differ significantly (*P*=.62; Figure 1F).

In addition to changes in influenza activity, we observed variations in environmental factors before and after the pandemic (Table 1). During the prepandemic period, AH, DTR, and RH were recorded at slightly higher levels (mean 7.64%, SD 5.20%; mean 11.38, SD 1.69 °C; and mean 61.16%, SD 8.85%, respectively) than in the postpandemic period (mean 6.22%, SD 4.77%; mean 10.82, SD 1.38 °C; and mean 56.01, SD 8.96%, respectively). Conversely, wind speed and visibility showed the opposite pattern, with higher values in the postpandemic period (mean 20.02, SD 3.09 km/h and mean 18.46, SD 1.03 km, respectively) than in the prepandemic period (mean 18.94, SD 2.88 km/h and mean 11.54, SD 1.76 km, respectively). However, certain climate factors, including cloud cover, apparent temperature, solar energy, solar radiation, temperature, UV index, and wind direction, did not show statistically significant differences across the examined periods, as indicated by the *t* test (*P*≥.05 in all cases).

**Figure 1.**
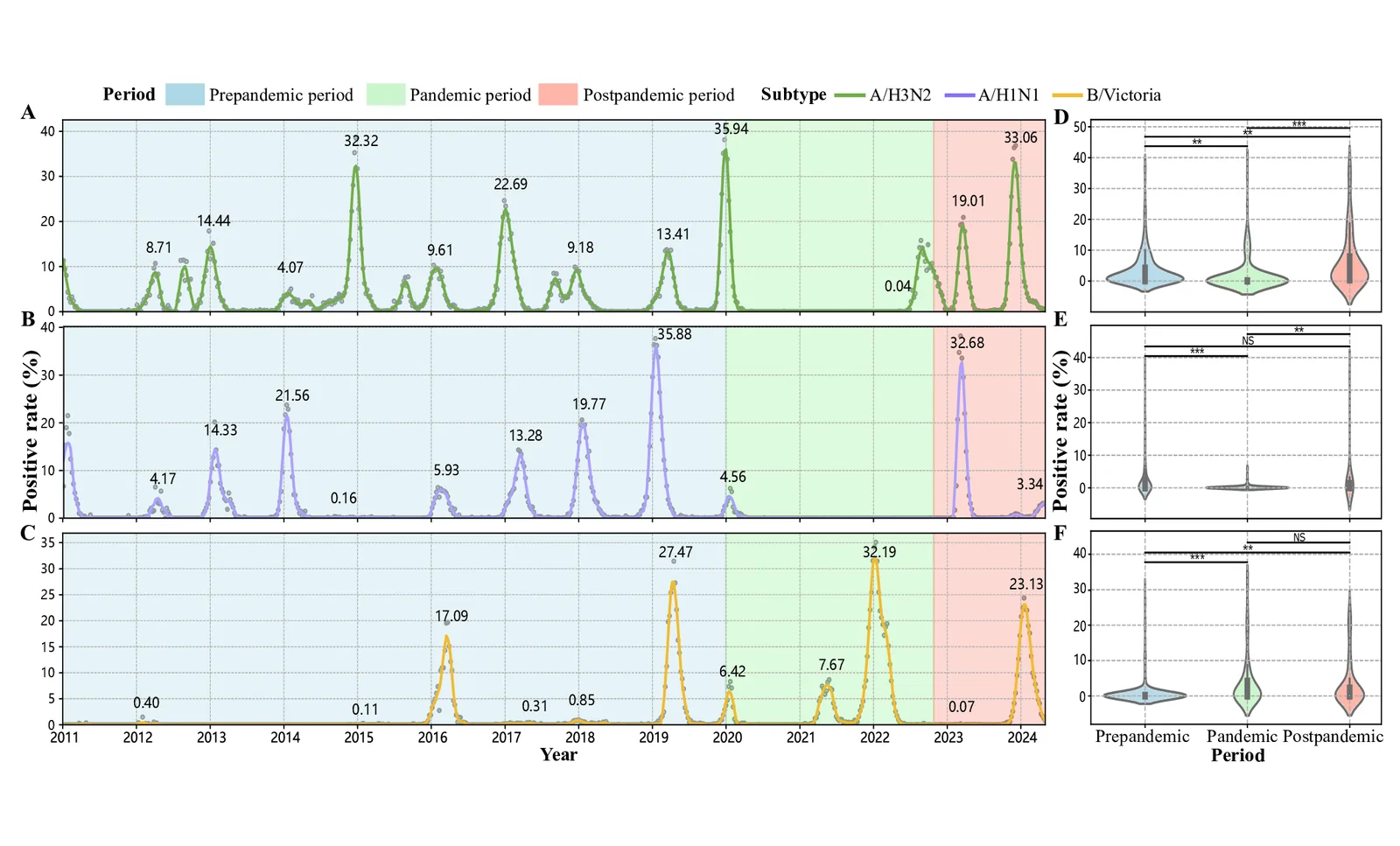
Temporal trends and distributions of influenza subtype positivity rates across study periods in northern China (2011-2024). (A-C) Temporal trends in the positivity rates of A/H3N2, A/H1N1, and B/Victoria, respectively. Background shading indicates the prepandemic, pandemic, and postpandemic periods. (D-F) Violin plots showing the distribution of positivity rates for A/H3N2, A/H1N1, and B/Victoria, respectively, across the 3 study periods. Horizontal brackets indicate pairwise comparisons between periods. *P<.05; **P<.01; ***P<.001. NS: not significant.

**Table 1.** Comparative analysis of environmental parameters between the pre- and postpandemic periods in northern China.

Environmental factors	*P* value	Period, mean (SD)		Period, median (IQR)	
		Prepandemic period	Postpandemic period	Prepandemic period	Postpandemic period
AH^a^ (%)	<.05^b^	7.64 (5.20)	6.22 (4.77)	6.29 (2.85 to 11.81)	4.65 (2.50 to 8.44)
Cloud cover (%)	.11	45.37 (10.92)	47.73 (12.13)	45.52 (37.74 to 53.54)	50.13 (37.62 to 56.50)
DTR^c^ (°C)	<.05^b^	11.38 (1.69)	10.82 (1.38)	11.27 (10.29 to 12.39)	10.88 (9.84 to 11.85)
Apparent temperature (°C)	.15	9.74 (12.90)	7.53 (12.62)	12.03 (−2.79 to 21.50)	7.62 (−4.43 to 17.57)
RH^d^ (%)	<.05^b^	61.16 (8.85)	56.01 (8.96)	61.30 (54.43 to 68.13)	53.93 (49.91 to 63.94)
SLP^e^ (mb)	.05^b^	936.06 (89.93)	1018.49 (8.86)	1004.85 (838.79 to 1018.81)	1019.17 (1011.22 to 1025.41)
Solar energy (MJ/m^2^)	.13	15.88 (5.07)	14.89 (5.38)	16.40 (10.85 to 20.48)	14.43 (9.55 to 19.88)
Solar radiation (W/m^2^)	.13	183.91 (58.74)	172.44 (62.27)	189.89 (125.55 to 237.22)	167.08 (110.55 to 230.19)
Temperature (°C)	.14	10.94 (11.22)	8.94 (11.10)	12.73 (0.08 to 21.57)	8.75 (−1.69 to 17.75)
UV index	.50	6.37 (1.39)	6.24 (1.58)	6.69 (5.06 to 7.56)	6.22 (4.69 to 7.61)
Visibility (km)	<.05^b^	11.54 (1.76)	18.46 (1.03)	11.71 (10.61 to 12.65)	18.63 (18.14 to 19.19)
Wind direction (degrees)	.54	180.02 (26.81)	181.92 (25.21)	180.12 (162.17 to 197.47)	182.78 (166.31 to 200.03)
Wind speed (km/h)	<.05^b^	18.94 (2.88)	20.02 (3.09)	18.54 (16.87 to 20.69)	20.17 (17.67 to 21.94)

a AH: absolute humidity.

b Statistical significance.

c DTR: diurnal temperature range.

d RH: relative humidity.

e SLP: sea level pressure.

### Environmental Determinants of Influenza Subtype Activity

We used XGBoost classification models to investigate the associations between environmental variables and influenza positivity rates for A/H3N2, A/H1N1, and B/Victoria subtypes in northern China. Detailed model results are summarized in Table S1 in Multimedia Appendix 1. To quantify the contribution and influence of each environmental feature on model predictions, we used the SHAP algorithm. Figure 2 illustrates the relative importance of 13 environmental factors for different influenza subtypes derived from SHAP values, with colors representing the Spearman correlation between influenza subtypes and climatic variables. The corresponding relative importance values and Spearman correlation coefficients are provided in Table S2 (Multimedia Appendix 1).

Our analysis revealed distinct patterns of climatic influence across the 3 influenza subtypes. Notably, AH emerged as the most important environmental factor associated with A/H3N2 and A/H1N1 activity, with SHAP contributions of 32.2% and 38.5%, respectively. In contrast, SLP showed the highest SHAP contribution (21.0%) for B/Victoria, followed closely by visibility (Figure S1 in Multimedia Appendix 1). To elucidate the dynamic variation in influenza positivity rates across pre- and postpandemic periods, we stratified the SHAP results into 2 phases (Figure S2 in Multimedia Appendix 1). This stratification revealed that AH remained an important environmental factor across all influenza subtypes, consistently ranking first in importance for both A/H3N2 and A/H1N1 before and after the pandemic. Interestingly, visibility and temperature assumed greater significance in the postpandemic period, particularly for A/H3N2 and A/H1N1. For A/H3N2, the importance of visibility increased significantly (rank: fourth before the pandemic vs second after the pandemic), showing a predominantly positive association with influenza positivity rates (Figure S2A in Multimedia Appendix 1). Similarly, for A/H1N1, the importance of visibility rose substantially (rank: fourth before the pandemic vs second after the pandemic), emphasizing its growing impact in the postpandemic phase (Figure S2B in Multimedia Appendix 1).

**Figure 2.**
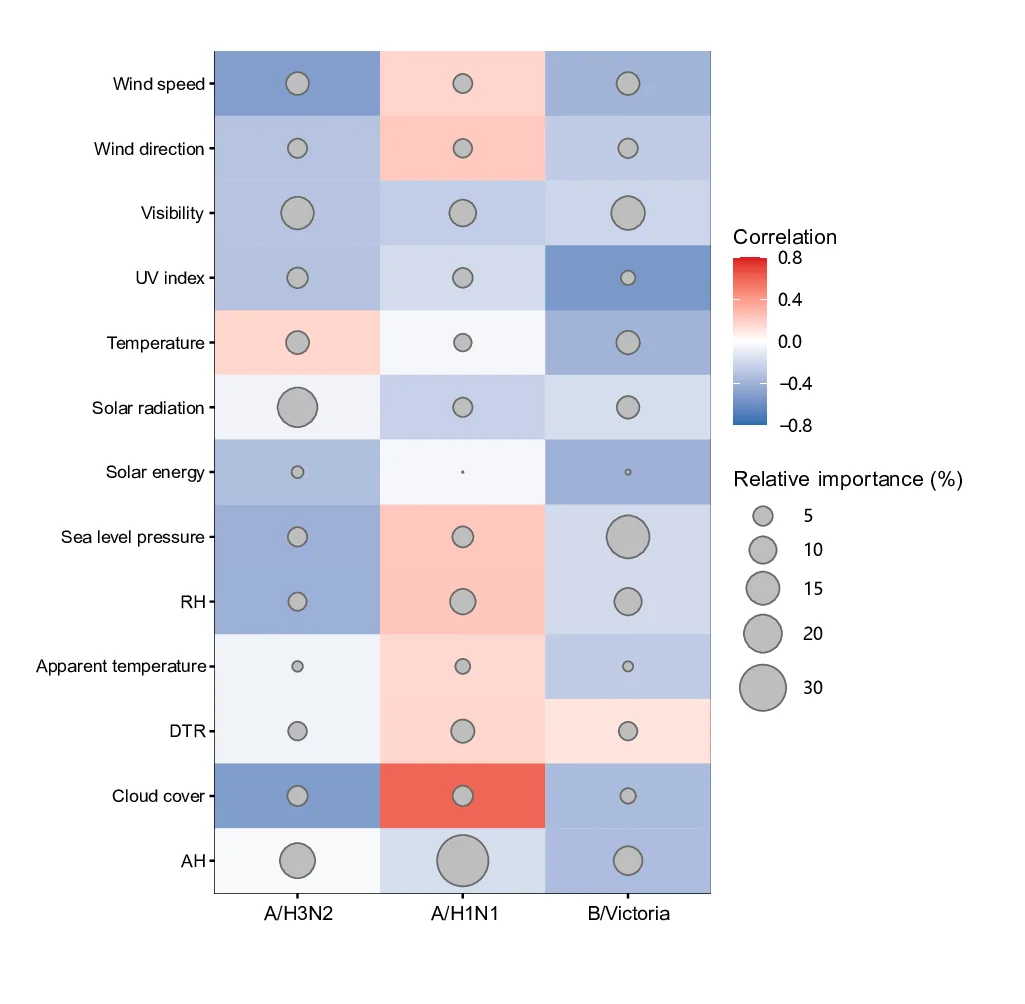
Correlation and relative importance of environmental factors for A/H3N2, A/H1N1, and B/Victoria. Associations between environmental factors and influenza subtype positivity rates were evaluated using Spearman correlation analysis and Extreme Gradient Boosting (XGBoost) models. Circle size represents the relative importance of each variable in the XGBoost model, and color represents the corresponding Spearman correlation coefficient. AH: absolute humidity; DTR: diurnal temperature range; RH: relative humidity.

We observed that several climatic variables, including wind speed, wind direction, SLP, RH, DTR, and cloud cover, exhibited opposing trends between A/H3N2 and A/H1N1. Notably, the importance of RH was lower than that of AH for A/H3N2 and A/H1N1 (4.9% and 10.7%, respectively) but higher for B/Victoria (12.4%). These findings suggest that AH showed a stronger association with influenza A subtypes, while RH appears more relevant for B/Victoria. SLP and visibility were among the most important environmental factors associated with the B/Victoria subtype before the pandemic, with the role of visibility becoming more pronounced after the pandemic (rank: second before the pandemic vs first after the pandemic). The increase in SHAP values for visibility after the pandemic suggests that visibility became a more influential environmental indicator associated with influenza activity (Figure S2C in Multimedia Appendix 1). Importantly, we noted that the medians of the visibility SHAP values shifted from negative to positive across all subtypes, indicating that the primary impact of visibility on influenza gradually transitioned from suppression to enhancement. We noted differential effects of DTR on subtype transmission. For A/H3N2, DTR was associated with reduced transmission (Spearman coefficient=−0.13), suggesting that stable temperatures might aid in controlling outbreaks of this subtype. Conversely, A/H1N1 and B/Victoria showed positive associations with DTR (Spearman coefficients=0.19 and 0.10, respectively). Temperature also exhibited subtype-specific effects, enhancing the spread of A/H3N2 while showing an opposite interaction with B/Victoria. These nuanced relationships underscore the complex interplay between environmental factors and influenza subtype dynamics in northern China, particularly in the context of pre- and postpandemic periods.

### Environmental Thresholds Governing Influenza Subtype Risk Transitions

The identified key environmental factors likely exert specific influences on the outbreak risk of multiple seasonal influenza subtypes. To elucidate these relationships and determine critical threshold points at which pandemic risk transitions occur across different phases (before and after the COVID-19 pandemic), we used GAMs to evaluate the effect relationships between these crucial climatic factors and influenza subtype positivity rates (Figure 3).

**Figure 3.**
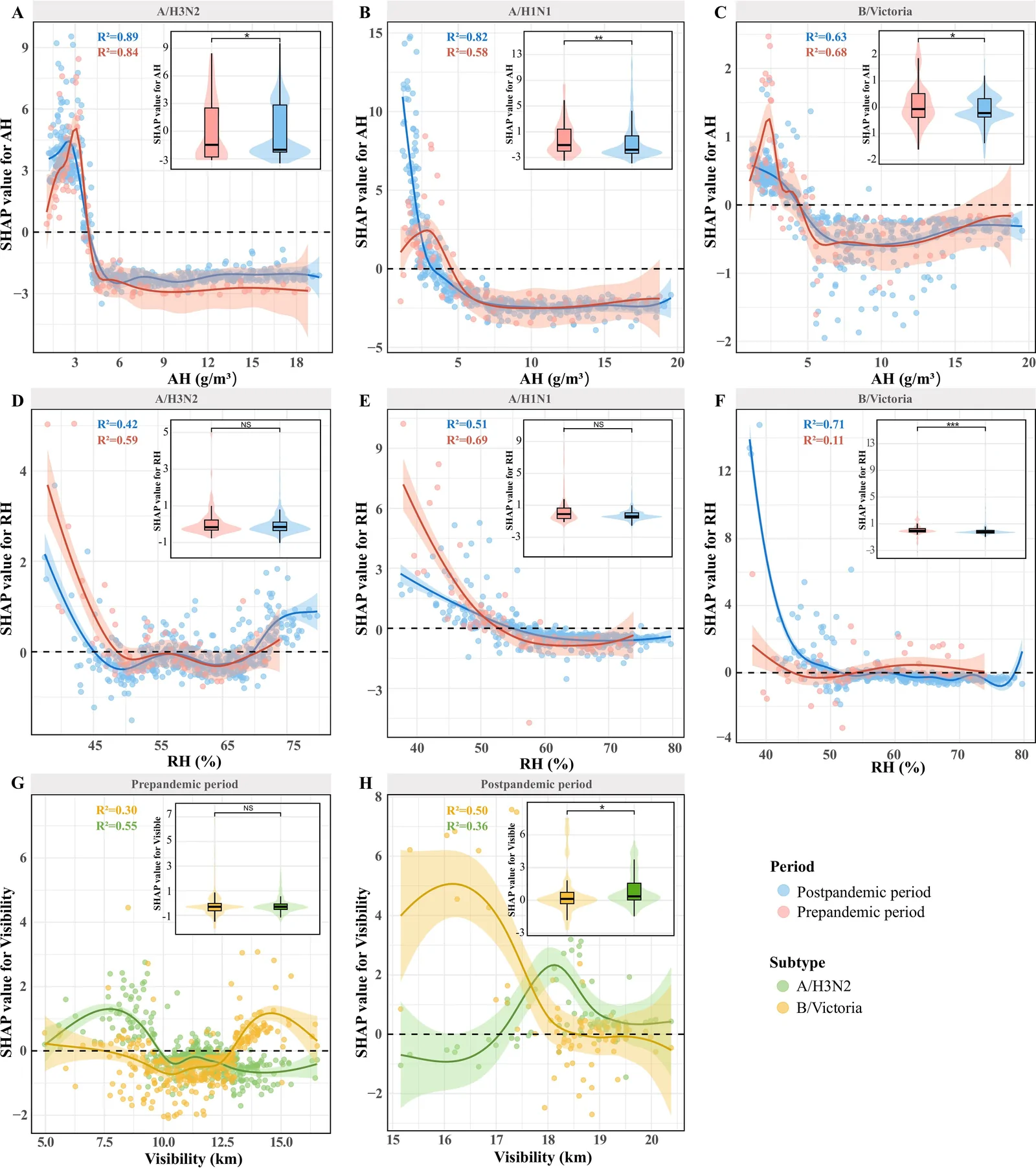
Relationships between Shapley additive explanations (SHAP) values and key environmental factors for A/H3N2, A/H1N1, and B/Victoria. (A-C) SHAP values for absolute humidity (AH) for A/H3N2, A/H1N1, and B/Victoria, respectively. (D-F) SHAP values for relative humidity (RH) for A/H3N2, A/H1N1, and B/Victoria, respectively. (G and H) SHAP values for visibility during the pre- and postpandemic periods, respectively. *P<.05; **P<.01; ***P<.001. NS: not significant.

Our GAM analysis revealed complex, nonlinear associations between key environmental variables and subtype-specific influenza positivity rates, identifying thresholds at which the direction of the model-estimated associations changed across different influenza strains. For A/H3N2, AH levels below 4 g/m^3^ were associated with positive SHAP values, indicating a positive association between low humidity conditions and positivity rates (Figure 3A). Above this threshold, SHAP values became negative, suggesting an inverse association between higher AH levels and A/H3N2 positivity. RH exhibited a complex nonlinear association with A/H3N2 positivity rates (Figure 3D). The GAM-estimated response pattern showed 2 thresholds where the direction of association changed: 45.1% (95% CI 43.5%-46.1%) and 69.7% (95% CI 69.2%-70.2%) in the prepandemic period and 48.7% (95% CI 46.7%-50.4%) and 70.6% (95% CI 66.3%-72.6%) in the postpandemic period. Between these thresholds, RH was predominantly associated with negative SHAP values, whereas RH levels outside this interval showed positive SHAP values, indicating a reversal in the direction of association. A/H1N1 positivity rates exhibited an L-shaped response to AH fluctuations, with the GAM curve crossing zero SHAP values at an AH threshold of 4.5 g/m^3^ (95% CI 4.0-4.8) during the postpandemic period (Figure 3B). For B/Victoria, a similar shift occurred at an AH threshold of 4.5 g/m^3^ (95% CI 4.0-4.8), with SHAP values showing an increasing trend at higher AH levels, particularly beyond approximately 12.5 g/m^3^ (Figure 3C). Conversely, A/H1N1 showed consistent declines in SHAP values in response to RH, with prepandemic cutoff points at 54.9% (95% CI 54.0%-55.6%) and postpandemic cutoff points at 52.8% (95% CI 52.0%-54.7%; Figure 3E). For B/Victoria, an RH level below 44.2% (95% CI 41.8%-71.5%) was associated with positive SHAP values during the prepandemic period, indicating a positive contribution to influenza positivity rates (Figure 3F).

Visibility exhibited distinct relationships with influenza subtypes, varying between pre- and postpandemic phases. In the prepandemic period, A/H3N2 showed an inverse curve relationship, initially rising then declining, with a shift from negative to positive impacts at a threshold of 9.69 km (Figure 3G). B/Victoria’s trend decreased initially and then gradually increased, surpassing zero at 12.9 km, peaking around 14 km before declining. After the pandemic, significant differences emerged; A/H3N2 exhibited a bell-shaped response, showing a negative association when visibility was below 17.1 km (95% CI 16.3-17.4), then gradually increasing to approximately 18.0 km before declining again (Figure 3H). For B/Victoria, the most pronounced positive influence on influenza positivity occurred in the visibility range of 15 to 17 km, diminishing and turning negative at 18.4 km (95% CI 18.0-18.9). These findings underscore the complex, subtype-specific interactions between environmental factors and influenza transmission dynamics, highlighting potential shifts in these relationships following the COVID-19 pandemic.

## Discussion

### Principal Findings

In this study, we used an interpretable machine learning framework to investigate the associations between environmental factors and influenza subtype activity during the pre- and postpandemic periods in northern China. This approach enabled us to characterize subtype-specific nonlinear association patterns, identify changes in the relative importance of environmental factors, and quantify SHAP-based turning points where the direction of associations changed across different environmental conditions.

Our analysis identified a notable shift in influenza dynamics, particularly with an increase in the activity of the B/Victoria subtype following the COVID-19 pandemic. The COVID-19 pandemic itself had profound effects on influenza epidemiology, leading to a shortened influenza season and decreased viral activity, as corroborated by previous studies [10,14,26]. PHSMs implemented to control COVID-19, such as enhanced use of personal protective equipment, reduced indoor gatherings, and strict physical distancing, likely contributed to these changes [7,10,27]. Our study particularly highlights the persistence of B/Victoria activity during and after the pandemic, in contrast to the fluctuations observed in influenza A subtypes. This could be attributed to differences in the affected populations: while influenza A subtypes are more prevalent among adults and older individuals—demographics also more susceptible to COVID-19—B/Victoria mainly affects children, who experienced less disruption from PHSMs and, therefore, continued to sustain transmission [28,29]. Thus, this may be the reason why this subtype accounted for most influenza cases (97.7%) during the COVID-19 pandemic in northern China [13]. Notably, upon the lifting of pandemic-related restrictions, A/H3N2 activity not only rebounded but exceeded prepandemic levels (*P*<.01), illustrating the dynamic nature of influenza transmission in response to changing public health measures. Additionally, we found that several environmental factors that are important to influenza transmission, such as AH and RH, also differed between the pre- and postpandemic periods. These alterations highlight the necessity of further investigation into the complex interplay between climatic factors and influenza transmission across different stages of the pandemic. Although SLP showed a relatively high contribution for B/Victoria in our model, this association should be interpreted cautiously. SLP is unlikely to represent a direct biological mechanism affecting viral characteristics; instead, it may reflect broader atmospheric conditions or correlated environmental factors that were not directly measured in this study.

Beyond the immediate effects of PHSMs, our study demonstrates that the influence of environmental factors on influenza transmission varies significantly by subtype and period. During the postpandemic period, while AH remained a significant determinant for influenza A subtypes (A/H3N2 and A/H1N1), its overall impact appeared reduced compared to the prepandemic phase, with SHAP values indicating a decrease in influence. This observation suggests that environmental changes following the pandemic may have altered the relative importance of different environmental determinants of influenza transmission. Interestingly, visibility, an indicator related to ambient air quality rather than a conventional meteorological variable, emerged as an important factor affecting all three influenza subtypes, particularly in the postpandemic period. Reduced visibility often reflects increased concentrations of airborne particulate matter and other pollutants, which may influence influenza transmission through effects on respiratory defense mechanisms and host susceptibility [30-32]. A previous study has also suggested that reduced visibility is associated with the risk of influenza infection, which is consistent with the findings of our study [33]. The increased importance of visibility after the pandemic may reflect broader environmental changes or shifts in population behavior and mobility, warranting further investigation into its role in influenza epidemiology.

We used GAMs to further investigate the complex, nonlinear relationships between environmental factors and influenza transmissibility, revealing variations across subtypes and periods. For example, the L-shaped relationship between AH and SHAP values for A/H3N2, A/H1N1, and B/Victoria suggests that influenza transmission is enhanced at lower AH levels but diminishes as AH increases, stabilizing beyond a certain threshold. Notably, the inflection points of the fitting curves at SHAP=0 consistently fell within the range of 3 to 5 g/m^3^, aligning with previous studies that indicated the onset of influenza seasons in temperate climates when AH drops below 5 g/m^3^ [34]. Higher AH levels have been shown to lead to the surface inactivation of lipid-containing viruses, potentially inhibiting the spread of influenza [35]. Similarly, the fitting curves for RH revealed that lower RH levels promote influenza transmission, but its influence diminishes and eventually transitions to a suppressive role at higher RH levels, particularly for A/H1N1. These findings underscore the importance of considering subtype-specific responses to environmental factors when developing predictive models for influenza transmission. Furthermore, our analysis of visibility revealed distinctive patterns for A/H3N2 and B/Victoria, with opposite S-shaped relationships observed between SHAP values and visibility levels. During the prepandemic period, low visibility initially promoted A/H3N2 transmission, while higher visibility had a suppressive effect. For B/Victoria, the relationship was more complex, with visibility initially suppressing and then promoting transmission as it increased. The postpandemic period saw an intensification of these effects, suggesting that changes in visibility, possibly linked to environmental or behavioral shifts, played a more prominent role in influenza transmission. The reasons for these subtype-specific responses to visibility are not entirely clear, but they may involve differences in viral sensitivity to environmental conditions or competition between subtypes [30,32,36]. Previous studies have highlighted the role of air pollutants, such as PM_2.5_ and PM_10_, in increasing the risk of influenza, particularly for influenza A subtypes, further supporting the need for a deeper exploration of visibility’s impact on influenza epidemiology [37].

### Limitations

Despite the valuable insights provided by this study, several limitations should be acknowledged. First, this study relied on passive surveillance data, which may be subject to measurement errors and inconsistencies in case reporting. Variability in surveillance practices, laboratory confirmation processes, and health care–seeking behaviors can introduce biases that may potentially affect the accuracy of our findings. Additionally, our analysis did not explore the potential interactions among different climatic factors, which could offer a more comprehensive understanding of how these variables collectively influence influenza transmission. Future studies should aim to investigate these interactions to provide a deeper insight into the complex dynamics involved. Moreover, the complex relationships between environmental variables and influenza activity necessitate more detailed spatial and temporal analyses. In this study, influenza surveillance data were aggregated across northern China, limiting our ability to evaluate spatial heterogeneity arising from differences in climatic conditions, demographic characteristics, and urbanization levels. In addition, meteorological factors may affect influenza activity with delayed effects due to viral survival, transmission processes, incubation periods, and health care–seeking behaviors; however, this study primarily focused on concurrent associations between environmental variables and influenza activity. Factors such as regional climatic zones, demographic characteristics, tourism activities, changes in population immunity, viral evolution, and PHSM implementation may create varied baseline transmission potentials [10,38,39]. These factors may influence the observed associations between environmental conditions and influenza activity in ways that are not fully captured by our study. Consequently, the observed differences between the pre- and postpandemic periods should not be interpreted as reflecting changes in the intrinsic effects of environmental factors alone. Therefore, further research is needed to disentangle these complex dynamics and assess how different environmental and behavioral factors contribute to influenza transmission across diverse geographic settings.

### Conclusions

This study provides a comprehensive analysis of the associations between environmental factors and influenza subtype activity in northern China during the pre- and postpandemic periods. We identified significant shifts in the relative importance of different environmental factors, with visibility emerging as a critical determinant of influenza transmission following the COVID-19 pandemic. AH remained an important environmental factor influencing influenza activity both before and after the pandemic, with its importance being the highest for influenza A and RH having a greater influence for the B/Victoria subtype. These findings underscore the importance of developing adaptive public health strategies that consider the changing environmental influences on influenza transmission, particularly as the world continues to face the long-term impacts of the COVID-19 pandemic. By integrating environmental data into public health planning, the effectiveness of influenza control measures can be enhanced, ultimately reducing the burden of seasonal and pandemic influenza on global health.

## Supplementary material

10.2196/91124 Multimedia Appendix 1 Model performance metrics for influenza subtypes A/H3N2, A/H1N1, and B/Victoria; relative importance and Spearman correlations of environmental factors across influenza subtypes A/H3N2, A/H1N1, and B/Victoria; Shapley additive explanations (SHAP) values for identifying important factors for prediction; SHAP values of the environmental factors during the pre- and postpandemic periods.

## Abbreviations

AH: absolute humidity
DTR: diurnal temperature range
GAM: generalized additive model
PHSM: public health and social measure
RH: relative humidity
SHAP: Shapley additive explanations
SLP: sea level pressure
XGBoost: Extreme Gradient Boosting
