# Supplementary material for: Climate-Driven Perturbations in Influenza Subtype Positivity Before and After the COVID-19 Pandemic: Observational Study
Source: JMIR Public Health Surveill. 2026 Nov 11;12:e91124. doi: 10.2196/91124 (PMC13754178; doi:10.2196/91124)
Supplement: Supplementary file 1 [file publichealth-v12-e91124-s001.docx]

**Supplementary information**

Climate-Driven Perturbations in Influenza Subtype Positivity Before and After the COVID-19 Pandemic: Observational Study

**Table of contents:**

**Table S1.** Model Performance Metrics for Influenza Subtypes H3N2, H1N1, and B/Victoria

**Table S2.** Relative Importance and Spearman Correlations of Environmental Factors Across Influenza Subtypes A/H3N2, A/H1N1, and B/Victoria

**Figure S1.** SHAP Values for Identifying Important Factors for Prediction

**Figure S2.** SHAP Values of the Environmental Factors during Prepandemic and Postpandemic Periods, respectively

**Table S1.** Model Performance Metrics for Influenza Subtypes

| Subtypes | MSE | RMSE | MAE | R^2^ |
| --- | --- | --- | --- | --- |
| A/H3N2 | 0.00188 | 0.04333 | 0.03023 | 0.99995 |
| A/H1N1 | 0.00033 | 0.01818 | 0.01287 | 0.99999 |
| B/Victoria | 0.00019 | 0.01368 | 0.00946 | 0.99999 |

**Table S2.** Relative Importance and Spearman Correlations of Environmental Factors Across Influenza Subtypes

| Environmental factor | Relative Importance (%) | | | Correlation | | |
| --- | --- | --- | --- | --- | --- | --- |
|  | A/H3N2 | A/H1N1 | B/Victoria | A/H3N2 | A/H1N1 | B/Victoria |
| AH | 32.24 | 38.53 | 11.28 | -0.10 | -0.10 | -0.36 |
| Cloud cover | 6.17 | 6.26 | 3.47 | -0.59 | 0.63 | -0.50 |
| DTR | 4.14 | 8.85 | 4.10 | -0.13 | 0.19 | 0.10 |
| Apparent temperature | 5.72 | 2.56 | 1.49 | -0.06 | 0.24 | -0.31 |
| RH | 4.88 | 10.67 | 12.44 | -0.56 | 0.28 | -0.20 |
| Sea level pressure | 5.84 | 5.08 | 20.97 | -0.56 | 0.34 | -0.20 |
| Solar energy | 1.30 | 0.00 | 0.02 | -0.43 | -0.05 | -0.55 |
| Solar radiation | 10.37 | 4.75 | 2.51 | -0.11 | -0.24 | -0.17 |
| Temperature | 6.75 | 2.68 | 8.40 | 0.15 | -0.04 | -0.54 |
| UV index | 5.14 | 1.47 | 6.14 | -0.39 | -0.22 | -0.63 |
| Visible | 7.36 | 9.93 | 17.80 | -0.31 | -0.31 | -0.25 |
| Wind direction | 6.63 | 2.60 | 6.04 | -0.32 | 0.39 | -0.31 |
| Wind speed | 3.45 | 6.62 | 5.34 | -0.59 | 0.20 | -0.54 |

**Figure S1.** SHAP Values for Identifying Important Factors for Prediction. (A) A/H3N2, (B) A/H1N1 and (C) B/Victoria

**Figure S2.** SHAP Values of the Environmental Factors during Prepandemic and Postpandemic Periods
